# Identification of a Novel Thermostable Alkaline Protease from *Bacillus megaterium*-TK1 for the Detergent and Leather Industry

**DOI:** 10.3390/biology9120472

**Published:** 2020-12-16

**Authors:** Tamilvendan Manavalan, Arulmani Manavalan, Shiyamsundar Ramachandran, Klaus Heese

**Affiliations:** 1 Centre for Advanced Studies in Botany, University of Madras, Chennai, Tamil Nadu 600 025, India; arulmanim@gmail.com; 2 Department of Biotechnology, Anna University, BIT-Campus, Tiruchirappalli, Tamil Nadu 620 024, India; shiyamsundarar@gmail.com; 3 Graduate School of Biomedical Science and Engineering, Hanyang University, 222 Wangsimni-ro, Seongdong-gu, Seoul 133-791, Korea

**Keywords:** *Bacillus megaterium*, detergent, hydrolase, protease, thermostable

## Abstract

**Simple Summary:**

In the current investigation, we describe the characteristic features of a novel *Bacillus megaterium* bacterium-derived protease with excellent thermostable enzyme activity under stringent alkaline conditions. The protease is highly compatible with various detergents and thus appears to be an eco-friendly additive for a variety of industrial applications.

**Abstract:**

An increased need by the green industry for enzymes that can be exploited for eco-friendly industrial applications led us to isolate and identify a unique protease obtained from a proteolytic *Bacillus megaterium*-TK1 strain from a seawater source. The extracellular thermostable serine protease was processed by multiple chromatography steps. The isolated protease displayed a relative molecular weight (MW) of 33 kDa (confirmed by zymography), optimal enzyme performance at pH 8.0, and maximum enzyme performance at 70 °C with 100% substrate specificity towards casein. The proteolytic action was blocked by phenylmethylsulfonyl fluoride (PMSF), a serine hydrolase inactivator. Protease performance was augmented by several bivalent metal cations. The protease tolerance was studied under stringent conditions with different industrial dispersants and found to be stable with Surf Excel, Tide, or Rin detergents. Moreover, this protease could clean blood-stained fabrics and showed dehairing activity for cow skin with significantly reduced pollution loads. Our results suggest that this serine protease is a promising additive for various eco-friendly usages in both the detergent and leather industries.

## 1. Introduction

Proteases, also known as peptidases, degrade proteins and peptides by hydrolyzing their peptide bonds, thereby producing shorter byproducts. They belong to a group of industrial proteins that account for ~60% of the total global market [1,2]. Based on the functional group present in the active site of proteases, these enzymes can be divided into seven major groups: (1) Aspartic proteases, (2) Asparagine peptide lyases, (3) Cysteine proteases, (4) Glutamic proteases, (5) Metalloproteases, (6) Serine proteases, and (7) Threonine proteases [1,2]. Proteases used in industry are often classified according to their active pH range as acidic, neutral, or alkaline proteases [1,3]. Among them, alkaline proteolytic proteins are deemed to be a key group of proteins because of their wide use in different industries, including those for agriculture, detergents, textiles, and leather [1,3,4,5].

While proteases have been studied across different types of microbes, including bacteria, fungi, and virus [6,7,8,9], *Bacillus* species (bacteria) are among the best resources for commercial applications of proteases. Detergent-compatible proteases have been widely used to remove stains efficiently and are key players in the cleaning and dehairing processes in the leather industry [9,10,11]. Conventional methods apply large amounts of lime and sodium sulfide and thus are not eco-friendly as these materials contribute to a significant amount of environmental pollution [1,5]. Proteases usually do not tolerate high temperature or surfactants, and thermostable proteases are not able to tolerate alkaline pH conditions or surfactants [4,9,12]. Moreover, thermostable alkaline proteases do not tolerate a variety of solvents, metal ions, or surfactants, thus significantly compromising their potential applications in the wider detergent industry [12,13]. Hence, it is of utmost interest to find an alternative protease that shows excellent performance towards pH values, temperatures, surfactant types, metal ions, and solvents for effective commercial applications.

To isolate alkaline-tolerant and thermostable protease producing microbes, we looked for prawn- and artemia-rich marine water locations. Our current investigation demonstrates the identification, isolation, purification, and specifications of a new thermostable alkaline protease from the *B. megaterium*-TK1 strain. The protease showed an excellent performance in view of pH values, temperatures, surfactant types, bivalent metal cations, and solvents. Moreover, this protease was evaluated in terms of its efficacy when used as a detergent additive, as a blood stain remover from fabrics, and for the dehairing of cow skin. Our data indicates that this *B. megaterium*-TK1-derived protease could be an ideal candidate in the eco-friendly detergent and dehairing industries.

## 2. Materials and Methods

### 2.1. Materials

Sephadex G-100, diethylaminoethyl cellulose (DEAE-C), and casein were bought from Sigma-Aldrich (St. Louis, MO, USA). Flash protein ladder was bought from the Gel Company (San Francisco, CA, USA) and other chemicals were acquired from HiMedia Laboratories Pvt. Ltd. (L.B.S. Marg, Mumbai, India). Commercial detergents were secured from a local supermarket.

### 2.2. Microbial Strains and Growth Conditions

Marine water probes were collected in prawn- and artemia-rich locations from Kelambakam (Tamil Nadu, India) and diluted serially with sterile water. These probes were smeared on skim milk agar plates (NaCl (0.5% *w*/*v*), skim milk (10% *w*/*v*), and agar (2.0% *w*/*v*)) and kept at 60 °C for 24 h. Among others, the novel identified proteolytic strain (by skim milk hydrolysis) was the bacterium *B. megaterium*-TK1 (16S rDNA gene sequence was deposited at GenBank (accession number EU586034)).

*B. megaterium*-TK1 was cultivated in a production medium composed of 0.5% (*w*/*v*) gelatin, 0.1% (*w*/*v*) lactose, 0.2% K_2_HPO_4_, 0.5% MgSO_4_.7H_2_O, 0.5% NaCl, and 0.5% CaCl_2_·2H_2_O, at pH 9.0 at 37 °C with shaking at ~200 rpm. Upon a culture period of 42 h, the culture supernatant (1 L) was obtained by centrifugation at 10,000× *g* at 4 °C for 15 min.

### 2.3. Protein Content Quantification and Protease Activity

The alkaline protease activity was determined, with some modification, as described previously [11]. Briefly, the reaction mixture was incubated at 70 °C for 1 h with the diluted enzyme (0.1 mL) and 1% (*w*/*v*) casein as substrate (0.9 mL, 50 mM Tris/HCl buffer, pH 8.0) and the reaction was terminated by adding 1 mL of 0.1 M trichloroacetic acid (TCA). Enzymatically hydrolyzed casein was measured using Folin-Ciocalteu reagent against inactive enzyme treated with casein as control. One unit of the enzyme performance was determined as the amount of enzyme needed to release 1 µmol of tyrosine in 1 h at 70 °C. The specific performance is represented in units/mg of protein. Protein quantification was estimated by the method of Bradford [14] or densitometry (absorbance at 280 nm).

### 2.4. Purification Procedure

Using the cleared culture supernatant (1 L), the protein precipitation was executed with 30–80% ammonium sulphate and the precipitate was dissolved in 10 mM Tris/HCl buffer (pH 8.0). Protein dialysis was performed with a 12 kDa cutoff membrane (Sigma-Aldrich, Cat. No. D6191-25EA) against the same buffer with three changes at 8 h intervals to remove excess ammonium sulphate and then lyophilized (Freeze Dryer, FD-10M, Labfreez Instruments Co., Ltd., Beijing, P.R. China). Further protein purification was performed via DEAE-C and Sephadex G-100 columns and fractionated (LKB Bromma 7000 Ultrovac fraction collector, Bromma, Sweden) as described previously [15]. Fractions exhibiting protease activity were finally stored in 10 mM Tris/HCl buffer (pH 8.0).

### 2.5. Sodium Dodecyl Sulphate-Polyacrylamide Gel Electrophoresis (SDS-PAGE) and Zymogram Analyses of Purified Protease

Standard SDS-PAGE procedures were performed to determine the molecular weight (MW) of the purified protease [16,17]. Zymography was performed as described by Rajkumar et al. [9]. Protein samples were loaded without boiling onto a zymogram gel substrate modified with 0.1% (*w*/*v*) bovine serum albumin (BSA). Upon completion of the SDS-PAGE, the gel was extensively washed (0.05 M Tris/HCl (pH 8.0) ± 2.5% Triton X-100; with frequent exchange of buffer) for 30 min to eliminate excess SDS. Thereafter, the gel was allowed to hydrolyze the BSA substrate in an incubation step for 1 h at 70 °C. Finally, the gel was stained with CBB R-250 to determine the presence of protease activity.

### 2.6. Effect of pH and Temperature on Protease Activity

The optimum pH for protease activity was measured using 1% casein as a substrate under different pH conditions using 50 mM solutions of the following buffers: citrate-phosphate (pH 3.0), sodium acetate (pH 4.0–6.0), Tris/HCl buffer (pH 7.0–9.0), glycine/NaOH (pH 10.0–11.0), and Na_2_HPO_4_/NaOH (pH 12.0). To study the pH stability, the protease was preincubated in the buffers and its activity was determined as described above [12].

The optimal temperature for the protease activity was determined by incubating the protease with 1% casein as the substrate at temperatures ranging from 20 to 100 °C for 1 h in 50 mM Tris/HCl (pH 9.0). Temperature-dependent protease stability was determined by pre-incubating the protease at temperatures of 20–100 °C for 1 h, followed by measuring its remaining activity as described above [12].

### 2.7. Protease Substrate Specificity

Various substrates, namely azo casein, bovine albumin, casein, egg albumin, gelatin, and hemoglobin, were prepared at 1% concentration in 50 mM Tris/HCl buffer (pH 8.0). Protease activity was measured to determine substrate specificity of the protease under standard assay conditions, as described in Section 2.3 [11].

### 2.8. Effect of Various Inhibitors, Metal Ions, Solvents, Surfactants, and Commercial Detergents on Purified Protease Activity

Purified protease activity was tested by pre-incubating it with 1 mM solutions of different inhibitors: dithiothreitol (DTT), ethylenediaminetetraacetic acid (EDTA), iodoacetate, 2-mercaptoethanol, and phenylmethylsulfonyl fluoride (PMSF). The metal cations (1 mM, mostly bivalent) tested were Ca^2+^, Co^2+^, Cu^2+^, Hg^2+^, Mg^2+^, Mn^2+^, Na^+^, and Zn^2+^. The solvents (10% v/v in 50 mM Tris/HCl buffer (pH 8.0)) tested were acetone, dimethyl sulfoxide (DMSO), ethanol, methanol, and isopropanol. Various surfactants (1% *w*/*v* or *v*/*v*) were tested, including cetyltrimethylammonium bromide (CTAB, a cationic surfactant), SDS, Triton X-100, and Tween-20. The industrial detergents (1% *w*/*v*) investigated were Ariel, Challenge, Henko, Rin, Surf Excel, and Tide. Protease activity was determined using 1% casein as a substrate for the various conditions. In the case of inhibitors, metal cations, solvents, surfactants, and industrial detergents, the absence of all of these additives in the final analysis was defined as 100% (control).

### 2.9. Blood Stain Removal and Cow Skin Dehairing Efficacy of Purified Protease

Clean cotton fabric square pieces (4 cm × 4 cm) were stained with blood collected from the local abattoir. The stained fabrics were allowed to dry, soaked in 2% formaldehyde for 30 min, followed by a water-washing step to remove excess formaldehyde. These blood-stained cotton fabric pieces were then incubated at 60 °C for 1 h while shaking (200 rpm) in a 250 mL Erlenmeyer flask containing a total volume of 50 mL buffer (50 mM Tris/HCl, pH 8.0) and one of the following additives: (1) No protease or detergent, as a control, (2) the protease (500 U/mL), (3) Surf Excel detergent (10 mg/mL), and (4) a combination of the protease (500 U/mL) and Surf Excel detergent (10 mg/mL). Upon washing and drying, the treated cotton fabric cloths were visually scanned for the extent of blood stain removal [9].

Cow skin was collected from the local abattoir and cut into three pieces (4 cm × 4 cm). The flesh side of the skin samples were treated with partially purified protease (1000 U) obtained from the DEAE-C column fractions at 50 °C in Tris/HCl buffer (50 mM, pH 8.0). The conventional method of chemical dehairing was also performed using 10% lime and 2.5% sodium sulfide. All samples were incubated for 12 h overnight. A control skin was prepared without any additives and all the treated skin samples were examined by visual comparison. The histological features of treated skins were analyzed under the microscope after hematoxylin and eosin staining, using previously reported methods [9]. After completing the dehairing process, the spent liquids were collected to determine biological oxygen demand (BOD), chemical oxygen demand (COD), total dissolved solids (TDS), and total suspended solids (TSS) using standard methods for the examination of water and wastewater, as described previously [18].

### 2.10. Statistical Analysis

The data obtained in this study were analyzed by Student’s *t*-test using SPSS software (IBM^®^ SPSS^®^ Statistics, Armonk, NY, USA)). Data are presented as mean ± standard deviation (SD).

## 3. Results

### 3.1. Screening and Identification of Protease Producing Bacterium

**Figure 1:**
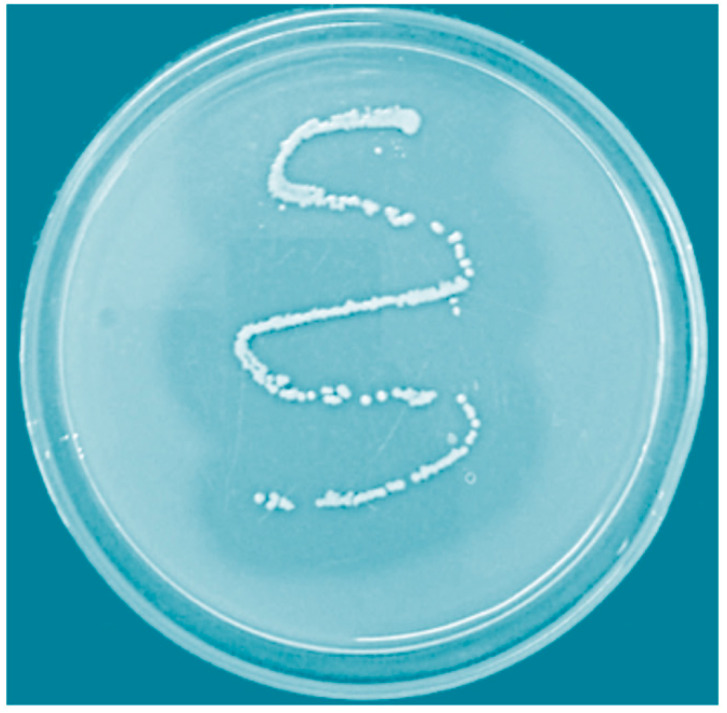
Screening of active protease release from *B. megaterium*-TK1 using skim milk agar plates. After 2 days of incubation, a clear transparent hydrolytic zone appeared against the white background when the plate was incubated with 10 mL of HgCl_2_ reagent (HgCl_2_ in 2 M HCl).

We screened prawn- and artemia-rich marine water locations to isolate protease producing microbes that were stable at alkaline pH and high temperature. The proteolytic activity of various candidates was screened on skim milk agar medium, and finally, *B. megaterium*-TK1 was selected as the most promising candidate. In general, after 24 h of incubation at 37 °C, the plates were added with 10 mL of HgCl_2_ reagent (HgCl_2_ in 2 M HCl) and incubated for 5–10 min. Next, the plates were gently rinsed twice with water. A clear hydrolytic zone of clearance was observed around the bacterial colonies (Figure 1), denoting its proteolytic activity (un-hydrolyzed proteins were precipitated).

### 3.2. Protease Purification

**Figure 2:**
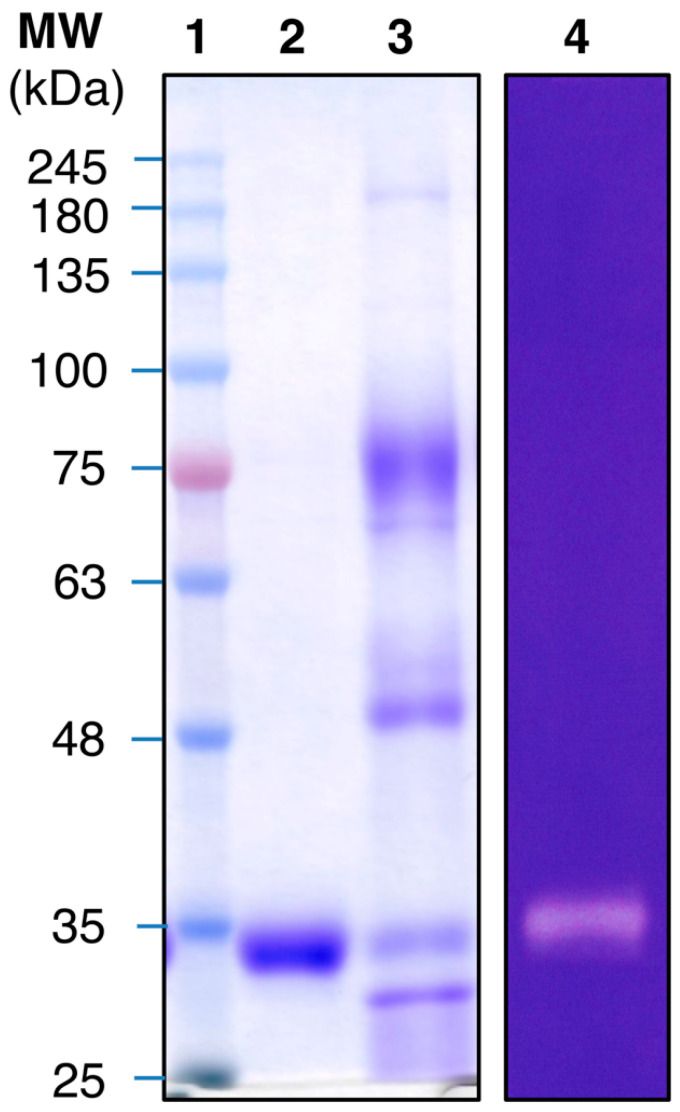
Molecular mass determination of purified protease from *B. megaterium*-TK1. SDS-PAGE analyses (lanes 1–3, stained with Coomassie Brilliant Blue R-250 (CBB R-250)) of isolated protease. Lane 1: pre-stained protein MW markers, lane 2: purified protease from Sephadex G-100 column chromatography (MW ~33 kDa), lane 3: partially purified protease from DEAE-C column chromatography. Zymography (lane 4) of purified protease on native-PAGE using 0.1% BSA as the substrate and stained with 0.1% (*w*/*v*) CBB R-250 (MW ~33 kDa).

Thereafter, an extracellular serine protease was purified from the culture filtrate (1 L) of this novel *B. megaterium*-TK1 after 48 h of culturing. The enzyme was purified using three major steps: initially, the crude enzyme (cell-free) was precipitated with 30–80% ammonium sulphate and subsequently was purified by a combination of DEAE-C and Sephadex G-100 gel filtration column chromatography. The summary of these protease purification steps from this novel *B. megaterium*-TK1 culture is presented in Table 1. The purified protease was isolated by Sephadex G-100 gel filtration column chromatography with a yield of 23.73% and a specific activity of 897 U/mg of protein. The obtained protease was subjected to SDS-PAGE and displayed a MW of ~33 kDa. Zymographic analysis (0.1% BSA as substrate) showed a white activity band of purified protease that was distinct against the blue background after staining with CBB R-250 and that corresponded to 33 kDa in native-PAGE (Figure 2).

### 3.3. Temperature- and pH-Dependent Protease Activity

**Figure 3:**
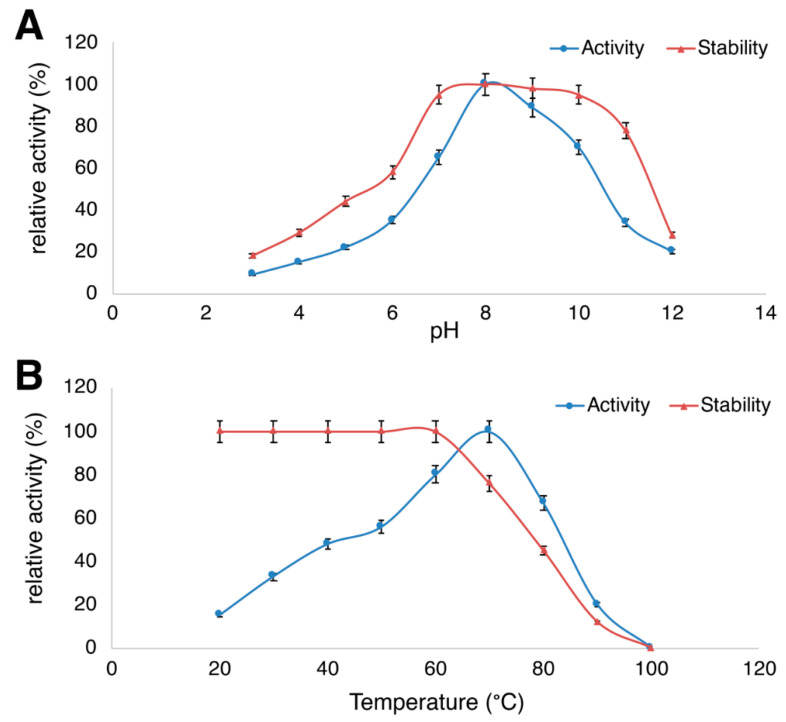
Effect of pH (**A**) and temperature (**B**) on *B. megaterium*-TK1-derived protease activity and stability. Values shown in (**A**) and (**B**) (mean ± standard deviation (SD)) are representative data obtained in triplicate tests from two independently performed experiments using purified *B. megaterium*-TK1-derived protease.

Further enzyme-characterizing analyses were performed, and the optimal activity of the purified protease was determined using different buffers (50 mM) with various pH ranging from 3 to 12. The enzyme was more active in alkaline pH, exhibited its best performance at pH 8.0 (in 50 mM Tris/HCl, pH 8.0, and 1% casein as substrate) and it was stable over a range of pH 7.0–11.0. The temperature-dependent activity of our isolated protease showed a performance-maximum at 70 °C (with 1% casein as the substrate). The thermal stability of our protease was maximum at 20–60 °C (in 50 mM Tris/HCl, pH 8.0), and it retained ~76% of its relative activity at 70 °C. However, upon incubating the protease above 70 °C, it decreased its activity abruptly (Figure 3).

### 3.4. Protease Substrate Specificity

Thereafter, we proceeded to check up on the enzyme’s substrate specificity. Our isolated protease showed effective hydrolysis towards casein (100%), whereas azo casein, bovine albumin, and hemoglobulin showed ~85%, 80%, and 79% hydrolysis, respectively. Egg albumin and gelatin hydrolysis were noticeably lower than other tested substrates (Table 2).

### 3.5. Impact of Various Inhibitors, Metal Cations, Solvents, and Surfactants on Purified Protease Activity

The protease activity was then further analyzed in the presence of different metal cations, inhibitors, surfactants, and solvents (Table 3). Our protease activity was completely blocked by PMSF, which suggested that it might be a serine-type protease. The protease activity was partially inhibited in the presence of DTT or 2-mercaptoethanol. Iodoacetate and EDTA did not inhibit protease activity significantly, thus suggesting that this enzyme is neither a cysteine protease nor a metalloprotease. Protease performance was also tested in the presence of different mono and bivalent metal cations. Among them, Na^+^ enhanced protease activity, followed by Mn^2+^, Ca^2+^, and Mg^2+^, while the effects of the other metal ions tested were insignificant. In the presence of Hg^2+^, protease activity was significantly inhibited. Protease activity was slightly enhanced by DMSO (*p* ≤ 0.017), whereas other solvents (acetone, ethanol, methanol, and isopropanol) did not significantly deteriorate the protease activity. The surfactants SDS and CTAB partially diminished the protease action, whereas Tween-20 and Triton-X 100 (non-ionic surfactants (*p* ≤ 0.01)) slightly enhanced the protease activity (Table 3).

### 3.6. Compatibility of Purified Protease Activity with Various Commercial Detergents and Protease Efficacy During Blood Stain Removal Procedures

**Figure 4:**
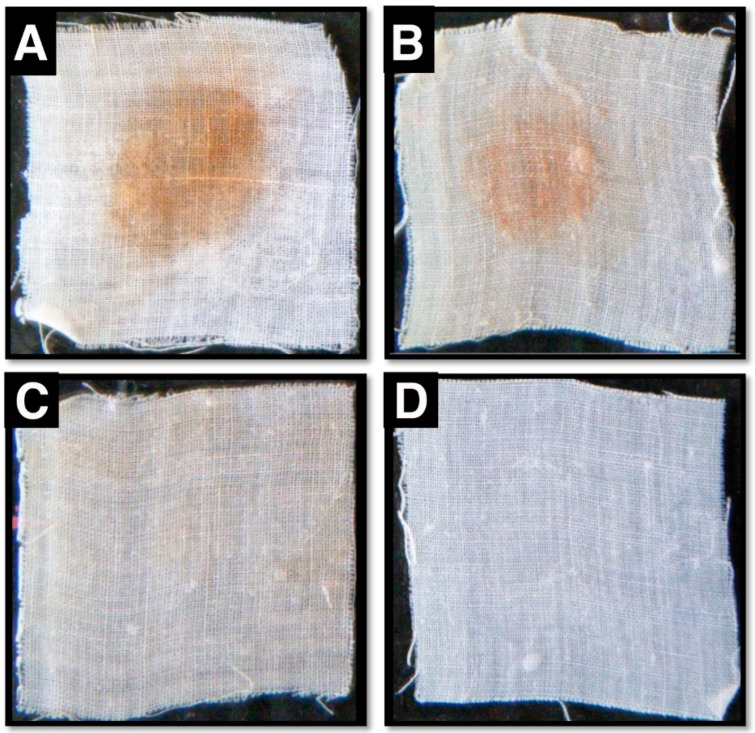
*B. megaterium*-TK1 protease-mediated cleaning performance of blood-stained fabrics. (**A**) Blood-stained fabric control. (**B**) Blood-stained fabric cleaned with detergent. (**C**) Blood-stained fabric cleaned with protease. (**D**) Blood-stained fabric cleaned with detergent and protease. Fotos were taken from cotton fabric square pieces (4 cm × 4 cm).

With regard to possible industrial applications, we analyzed the enzyme’s compatibility with various commercial detergents. The purified protease exhibited significant activity among the different commercial laundry detergents tested. We incubated the enzyme with different industrial laundry detergents for 1 h and observed that our enzyme retained up to 79–99% of its activity under these stringent conditions, with a maximum efficacy in the presence of Surf Excel, followed by Tide, Rin, Ariel, Challenge, and Henko, respectively (Table 4). While the protease alone decolored the blood spot from fabrics quite effectively, maximum washing performance efficiency was achieved when the protease was applied along with the commercial laundry detergent Surf Excel (Figure 4).

### 3.7. Dehairing of Cow Skin by Chemical Processing or Treatment with Purified Protease

**Figure 5:**
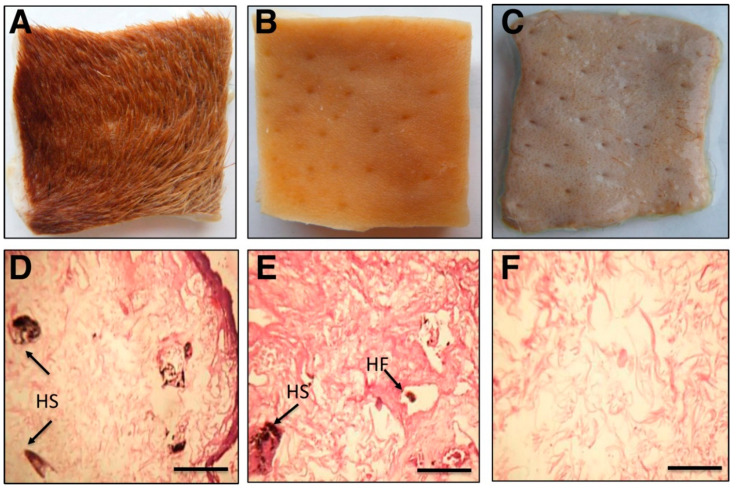
Dehairing efficiency of cow skin mediated by *B. megaterium*-TK1-derived (partially purified) protease or chemical treatment. (**A**) Cow skin with hair as control. (**B**) Chemical treatment of cow skin. (**C**) Enzymatic treatment of cow skin. (**D**) Cow skin with hair as control. (**E**) Chemical treatment of cow skin. (**F**) Enzymatic treatment of cow skin. (**D**–**F**) are photomicrographs from (**A**–**C**) samples at 100× magnification of a longitudinal cross-section of cow skin. Chemical treatment of cow skin still retained hair shafts (HS) and hair follicles (HF). Fotos were taken from collected cow skin pieces (4 cm × 4 cm, (**A**–**C**)); scale bar = 250 μm (**D**–**F**).

Additional industrial applications were considered, such as dehairing of cow skin. Cow skin dehairing was achieved with the partially purified protease by overnight incubation. After the enzymatic treatment, the skin hairs were removed smoothly by pulling with forceps. Compared with the chemical-treated cow skin, the hairs were much easier to remove upon treatment with the protease. The efficacy of protease and chemical treatment on cow skin hair removal was further examined by histological studies. The microscopic investigation showed that upon chemical treatment, hair shafts and hair follicles were retained in the skin compared to enzymatic treatment (Figure 5). Additionally, enzymatic treatment of cow skin showed considerably less pollution loads (Table 5).

## 4. Discussion

Here, we isolated, purified, and investigated the characteristic features of a novel protease from *B. megaterium*-TK1 and analyzed its possible industrial usefulness. Previously, several proteases were characterized from *Bacillus* spp. However, in terms of industrial applications, the commercial aspects of such proteolytic enzymes, particularly their toleration of pH, temperature, solvents, and surfactants, as well as their compatibility with commercial detergents, need to be established in more detail. In general, the MWs of proteases from various *bacillus* spp. were 25–71 kDa and are summarized in Table 6. The purified protease from our study had a MW of about 33 kDa in SDS-PAGE after Sephadex G-100 gel chromatography (yield: 23.73%, specific activity: 897 U/mg of protein). Asker et al. [19], Rajkumar et al. [9], and Yossana et al. [20] presented proteases from *B. megaterium* between 25 and 28 kDa (Table 6).

One of the interesting, key features of the protease in this work is its excellent performance under stringent alkaline pH (7.0–11.0) and temperature (20–70 °C) conditions, even after pre-incubation for 1 h, and an optimal performance activity at a quite high pH and temperature (pH 8.0, 70 °C). Compared to similar proteases reported from *Bacillus* spp. (Table 6, [31,36]), which are stable within a similar pH and temperature range, our protease exhibited optimal activity at the highest temperature among all *Bacillus* spp. proteases and thus was of utmost interest for eco-friendly industrial applications. The substrate specificity for each protease varied based on its type and binding energy. Our protease has maximum enzymatic activity for casein, which was in accordance with other *Bacillus* spp. protease reports [2,3,15,36,38].

It is well known that PMSF can modify the essential serine and even other amino acid residues (e.g., arginine, lysine, histidine, and tryptophan) in a protease-active substrate binding site, which results in complete loss of protease activity [9,12,36]. We noticed that our protease was highly sensitive to PMSF (complete inhibition at 5 mM PMSF, Table 3), indicating that our isolated enzyme was a serine-type protease. In the presence of different metal cations, our protease activity either enhanced (Na^+^, Mn^2+^, Ca^2+^, Mg^2+^, and Co^2+^) or remained constant (Cu^2+^ and Zn^2+^), except for mercury (Hg^2+^), which agreed with previous reports [9,12,36]. Our protease performed very well in the presence of various solvents (Table 3). Different solvents did not seriously deteriorate our protease performance, whereas DMSO slightly enhanced the protease activity. Similarly, other solvent-tolerant proteases were reported with different solvents at a concentration of 50% [3,31,39]. Thus, these proteases could also be useful in other industrial applications, such as alternative active biocatalysts for peptide synthesis in nonaqueous solvents [39].

The investigation of the impact of different surfactants revealed that ionic surfactants (SDS and CTAB) to some extent derogated the action of our protease, while non-ionic surfactants (Triton X-100 and Tween-20) slightly increased our protease action. Previous reports showed that non-ionic surfactants could either slightly enhance [3,36,38] or decrease (if applied at higher concentrations (e.g., Triton X-100 or Tween-20)) [2,3,36] the protease activity.

Another pivotal aspect for the possibility of industrial application is the compatibleness of the protease with industrial detergents and salts, and its ability to withstand harsh environmental conditions [23]. Our current protease showed excellent performance in the presence of a variety of commercially available laundry detergents (Surf Excel, Tide, Rin, Ariel, Challenge, and Henko) and retained its activity up to 79–99% after a 1 h incubation, similar to other *Bacillus* spp.-derived proteases [9,23]. Moreover, our current protease performed well in the presence of the bivalent cation Ca^2+^ and even showed an elevated protease activity, which might add an advantage considering water hardness during washing. The washing performance of our protease with blood-stained fabric cloths was very promising. Some reports observed that such protease treatment alone still left some unremoved stains due to the lack of efficient protease action in the presence of detergents [4,12]. Other reports observed that their proteases could clean fabrics in the presence of detergents [9,23]. Our protease could remove the stain alone (without the Surf Excel detergent) and very efficiently with the detergent and thus it would be an ideal candidate for such industrial applications.

The leather processing industry has a large environmental impact on earth, involving pollutants such as solid waste, lime, sulfide, and chromium [5,10]. Therefore, the dehairing processes using proteases are considered as an alternative green approach to reduce pollution loads compared to conventional chemical methods [5,11]. Earlier studies reported that alkaline proteases are promising in the dehairing of goat, sheep, buffalo, and cow skins [10,11,40,41]. Likewise, our partially purified protease from *B. megaterium*-TK1 was highly efficient in dehairing cow skin. Interestingly, the histological studies showed no retaining of hair shafts and hair follicles by our protease treatment. Moreover, the pollution loads from the protease cow skin treatment were less for BOD, COD, TDS, and TSS by 65.2%, 77.6%, 84.2%, and 87.6% respectively, compared to chemically treated methods. Thus, while maintaining the natural structure of the leather, our *B. megaterium*-TK1-derived protease appears to have a highly efficient dehairing ability without unnecessarily polluting the environment, making it an ideal candidate for the leather processing industry.

## 5. Conclusions

In this study, we presented a novel thermostable alkaline serine protease derived from a novel *B. megaterium*-TK1 strain. Our purified protease had an excellent performance under stringent conditions in terms of pH, temperatures, solvents, bivalent metal cations, surfactants, and commercial detergents. Our protease-supported washing performance and leather processing results were very notable in their ability to obtain clean fabrics and better quality of leather, while significantly decreasing pollution loads compared to chemical treatments. Hence, this *B. megaterium*-TK1-derived serine protease could be an ideal enzyme suitable for eco-friendly detergents and leather processing.

## Figures and Tables

**Table 1:** Overview of *B. megaterium*-TK1-derived protease purification steps and yields.

Purification Step	Total Protein (mg)	Total Activity (Units)	Specific Activity (Units/mg Protein)	Purification Fold	Yield (%)
Culture supernatant	62	22,666	366	1	100
(NH_4_)_2_SO_4_ Fraction 30–80% (*w*/*v*)	25	16,250	650	1.77	71.69
DEAE-C	10	7250	725	1.98	31.98
Sephadex G-100	6	5380	897	2.45	23.73

**Table 2:** Overview of substrate-dependent protease activity.

Substrate (1%)	Relative Activity (%)
Azo casein	85 ± 0.5
Bovine albumin	80 ± 3
Casein	100 ± 1
Egg albumin	49 ± 1.8
Gelatin	35 ± 1.5
Hemoglobin	79 ± 1

Mean values are from three independent experiments (maximum mean deviation ± 5%).

**Table 3:** Impact of different inhibitors, metal cations, solvents, and surfactants on the protease performance.

Chemicals (Concentration)	Relative Activity (%)
Control (none)	100 ± 0.1
Inhibitors (5 mM)	
DTT	67 ± 1.6
EDTA	90 ± 1.88
Iodoacetate	85 ± 1.0
2-mercaptoethanol	71 ± 0.5
PMSF	0.0 ± 0.0
Metal cations (1 mM)	
Control	100 ± 0.1
Ca^2+^	120 ± 1.9
Co^2+^	105 ± 2.5
Cu^2+^	95 ± 0.6
Hg^2+^	20 ± 1.0
Mg^2+^	111 ± 0.8
Mn^2+^	123 ± 2
Na^+^	155 ± 1.85
Zn^2+^	85 ± 1.5
Solvents (10%)	
Acetone	100 ± 0.9
DMSO	109 ± 4.0
Ethanol	95 ± 3.0
Methanol	88 ± 1.4
Isopropanol	90 ± 0.7
Surfactants 1% (1 h)	
Control	100 ± 0.1
CTAB	71 ± 1.5
SDS	69 ± 0.6
Triton X-100	109 ± 1.7
Tween-20	105 ± 0.9

Mean values are from three independent experiments (maximum mean deviation ± 5%).

**Table 4:** Detergent compatibility of the purified protease activity.

Detergents (1% *w*/*v*)	Relative Activity (%)
Ariel	89 ± 1.2
Challenge	85 ± 0.2
Henko	79 ± 0.5
Rin	93 ± 0.3
Surf Excel	99 ± 1.0
Tide	97 ± 0.5

Mean values are from three independent experiments (maximum mean deviation ± 5%).

**Table 5:** Comparison of pollution load generated during cow skin dehairing by chemical processing and treatment with the partially purified protease.

Pollution Loads (PPM)	Chemical Treatment	Enzymatic Treatment	Pollution Reduction (%)
BOD	2130 ± 50	1390 ± 20	65.2
COD	5795 ± 30	4500 ± 25	77.6
TDS	17,500 ± 40	14,750 ± 50	84.2
TSS	5650 ± 60	4950 ± 10	87.6

Mean values are from three independent experiments (maximum mean deviation ± 5%). Biological oxygen demand (BOD), chemical oxygen demand (COD), total dissolved solids (TDS), and total suspended solids (TSS).

**Table 6:** Comparison of physicochemical properties of various proteases from *Bacillus spp.* with the purified protease from *B. megatarium*-TK1.

Organism	Molecular Weight (kDa)/pI *	Optimal Activity at pH/Temperature (°C)	Stability at pH/Temperature (°C)	Reference
*B. megaterium* RRM2	27/–	10/60	7–11/60	[9]
*B. megaterium*	28/–	7.5/50	7.0–8.5/80	[19]
*B. megaterium*	27/–	10/50	7.5–9.5/30–45	[20]
*B. megaterium*	25/–	7.5/50	7.0–8.5/80	[19]
*B. megaterium*-TK1	33/–	8/70	7–11/20–60	present study
*B. altitudinis* W3	37.90/8.67	9.5/55	6.5–11.5/50	[21]
*B. altitudinis* W3	37.29/6.15	8.5/50	6–10.5/40	[21]
*B. altitudinis* W3	34.94/7.61	10.5/45	10.5–11.5/50	[21]
*B. alveayuensis* CAS 5	33/–	9/50	8–11/80	[22]
*B. aerius* NSMk2	9/–	8/45	6.5–9.5/65	[23]
*B. amyloliquefaciens* SYB-001	36.8/–	7/50	6–10/60	[24]
*B. halodurans* C-125	28.3/9.47	12/60	12/50	[25]
*B. invictae*	–/–	9–11/60	6–12/30–50	[26]
*B. koreensis* BK-P21A	48/–	9/60	7–10/70	[27]
*B. safensis* S406 (BS1)	29/–	11/60	6.0–12.0/45	[28]
*B. safensis* CK	40/–	7/37	–	[29]
*B. sp.* SB12	41/–	9/37	7–11/60	[30]
*B.* sp. SM2014	71/–	10/60	7–12/80	[31]
*B. subtilis* BP36	40/–	9/60	9–11/60	[32]
*B. subtilis* DR8806	37/–	8/45	6–9/60	[33]
*B. subtilis* GA CAS8	41/–	9/50	9–11/60	[34]
*B. subtilis* KT004404	28.2/–	6/55	5–8/65	[35]
*B. velezensis* SW5	34/4.66	8/40	6–9/20–40	[36]
*B. zhangzhouensis*	42/–	9.5/60	7–10.5/30–70	[37]

* pI = pH at the isoelectric point of the protein.
